# Applications of thin-film sandwich crystallization platforms

**DOI:** 10.1107/S2053230X16004386

**Published:** 2016-03-24

**Authors:** Danny Axford, Pierre Aller, Juan Sanchez-Weatherby, James Sandy

**Affiliations:** a Diamond Light Source, Harwell Oxford, Didcot OX11 0DE, England

**Keywords:** protein crystallization devices, *in situ* X-ray analysis, crystallization, crystal visual inspection, diffraction data collection

## Abstract

Examples are shown of protein crystallization in, and data collection from, solutions sandwiched between thin polymer films using vapour-diffusion and batch methods. The crystallization platform is optimal for both visualization and *in situ* data collection, with the need for traditional harvesting being eliminated. In wells constructed from the thinnest plastic and with a minimum of aqueous liquid, flash-cooling to 100 K is possible without significant ice formation and without any degradation in crystal quality. The approach is simple; it utilizes low-cost consumables but yields high-quality data with minimal sample intervention and, with the very low levels of background X-ray scatter that are observed, is optimal for microcrystals.

## Abstract 2

Crystallization *via* sandwiches of thin polymer films is presented and discussed.

## 1. Introduction   

The production of diffraction-quality crystals suitable for structure determination remains a significant hurdle in structural biology research. In many cases samples are lost or compromised by the action of crystal mounting and cryocooling for data collection. Attempts have been made to automate the crystal-fishing step (Cipriani *et al.*, 2012 ▸; Wagner *et al.*, 2013 ▸; Heidari Khajepour *et al.*, 2013 ▸); however, these approaches typically rely on expensive hardware and robotics which still work in a serial manner. Another approach is to transfer sample en masse to a device with high X-ray transparency optimized for data collection (Roedig *et al.*, 2015 ▸; Murray *et al.*, 2015 ▸); however, any sample-transfer step inevitably brings a risk of sample damage or loss. Collecting data *in situ* neatly sidesteps these problematic and labour-demanding procedures (Axford *et al.*, 2012 ▸; le Maire *et al.*, 2011 ▸; Bingel-Erlenmeyer *et al.*, 2011 ▸). Progress in this area has also been driven by novel designs of crystallization platforms that have been configured for *in situ* data collection, including microbatch under oil *via* the X-CHIP (Kisselman *et al.*, 2011 ▸), capillary arrays (Crystal*Harp* ^TM^) and designs of SBS microtitre plates in 96-well format (CrystalQuick^TM^X, MiTeGen *In Situ*-1^TM^, CrystalDirect^TM^). In parallel, the opportunity to collect data *in situ*, in addition to from individually looped samples on SPINE (or other) standard pins, is now offered by many facilities (for a review, see Aller *et al.*, 2015 ▸). As a result of this progress, there have been examples of structure determination *via in situ* data collection for what in the past would have been considered challenging targets (Axford *et al.*, 2015 ▸; Huang *et al.*, 2015 ▸; Wang *et al.*, 2012 ▸). Despite these examples, the majority of experimenters continue to work with crystallization platforms that are not fully optimized for *in situ* data collection, with the expectation that the samples will need to be extracted before high-quality X-ray data can be obtained. A particular area of crystallization driving the move to *in situ* data collection is the lipidic cubic phase or *in meso* phase (Caffrey, 2015 ▸). With this method, the issues associated with crystal fishing are amplified: the traditional glass-sandwich platform makes access to the samples problematic and the high viscosity of the lipid medium makes it impossible to isolate the sample completely from unwanted material that both obscures visualization of the sample and contributes significantly to background X-ray scatter. The act of replacing the glass with a thin polymer allows high-quality diffraction data to be collected without the need to remove samples from their point of origin within the crystallization platform (Huang *et al.*, 2015 ▸, 2016 ▸).

**Figure 1:**
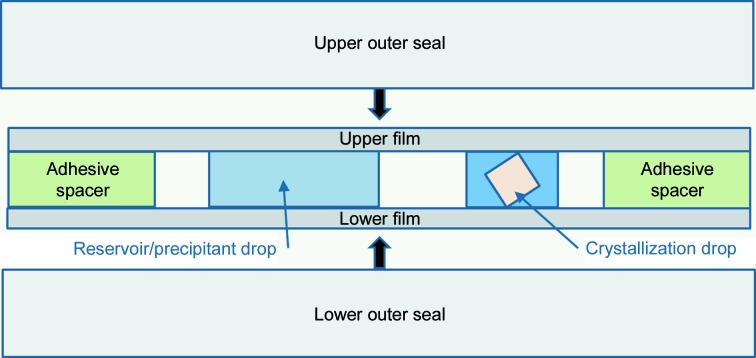
A schematic of a thin polymer-film sandwich plate for crystallization *via* vapour diffusion. An outer seal is designed to arrest evaporation and also to keep the film clean for optimum imaging; it is to be removed before data collection.

The work documented here exploits a platform, analogous to that described for *in meso* crystallization above, consisting of a thin polymer-film sandwich (TPFS) and applies it to crystallize soluble protein *via* vapour-diffusion and batch methods. A schematic of the construction of a TPFS-type plate for a vapour-diffusion experiment is shown in Fig. 1 ▸. Factors such as film and sandwich thickness have been explored and are discussed. Lysozyme was used to demonstrate proof of principle, its long-standing use making it a common metric for novel data-collection platforms and approaches. We found that it was possible to perform data collection *in situ* at room temperature and cryogenic temperatures using the same sample. Our findings demonstrate that the method provides a flexible and effective route to structural analysis without the need to transfer or physically manipulate crystals from their place of origin.

## 2. Materials and methods   

**Figure 2:**
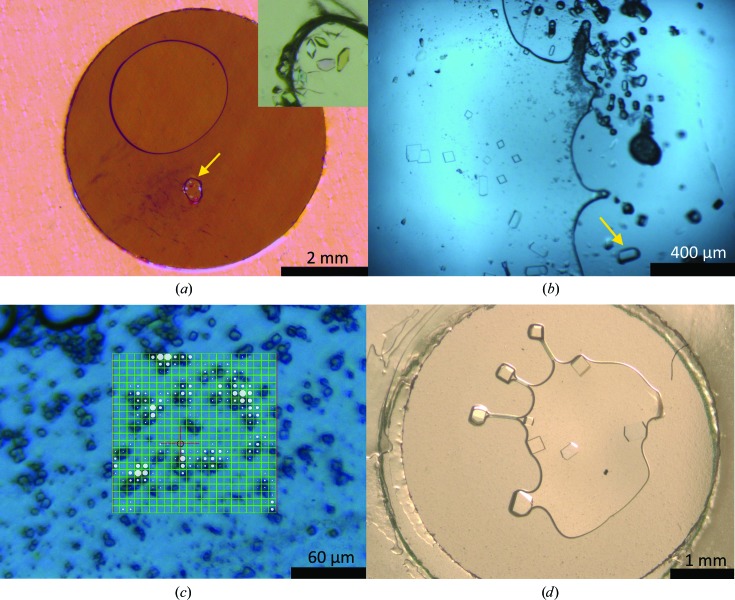
(*a*) A polarized light microscope image of a vapour-diffusion experiment dispensed with a Mosquito robot; the arrow indicates the protein-containing drop. Inset, an enlarged view of the drop with the lysozyme crystals clearly visible. (*b*) Lysozyme crystals grown *via* the batch method, dispensed by hand, in an in-house TPFS with a 10 µm spacer. The larger crystals within the drop on the left of the image have grown to fill the void of the sandwich. A number of crystals are visible on the right isolated from the mother liquor where the drop edge has receded. The yellow arrow indicates the crystal from which the diffraction data presented in §[Sec sec3.5]3.5 were collected. (*c*) An image of an in-house TPFS plate containing many lysozyme microcrystals captured through the I24 beamline-viewing system with diffraction spot-finding results of a grid (raster) scan overlaid by the *GDA* data-collection software. (*d*) A microscope image of lysozyme crystals grown *via* the batch method in a TPFS constructed with a 96-well spacer of 100 µm. The plate had been left for 4 d without a secondary seal and evidence of evaporation is clear, leaving crystals as ‘peninsulas’ from the drop. The crystals can be seen to form flattened cuboids, since growth is confined to the internal dimensions of the sandwich.

Commercial lyophilized lysozyme from chicken egg white (Sigma–Aldrich; CAS No. 2650-88-3) was resuspended in high-purity (18.2 MΩ cm) water at a concentration of 73 mg ml^−1^. Either batch or vapour-diffusion methods were used to grow lysozyme crystals. For the batch method, 100 nl lysozyme solution was mixed with 1 µl crystallization solution [20%(*w*/*v*) polyethylene glycol (PEG) monomethyl ether (MME) 5000, 1 *M* NaCl, 50 m*M* sodium acetate pH 4.6]. Different ratios of protein solution to crystallization solution were also explored with the intention of nucleating a large number of microcrystals (examples are shown in Figs. 2 ▸ *b*, 2 ▸ *c* and 2 ▸ *d*). For the vapour-diffusion method, drops were made by mixing 100 nl lysozyme solution with 100 nl reservoir solution [20%(*w*/*v*) PEG MME 5000, 1 *M* NaCl, 50 m*M* sodium acetate pH 4.5] and were equilibrated against 1 µl reservoir solution (an example is shown in Fig. 2 ▸ *a*).

TPFS crystallization plates were constructed in one of three ways. Firstly, viewdrop III hanging-drop cover film (TTP Labtech, Cambridge, England) was used as the lower film onto which liquid was dispensed, and a cover film was then attached by hand using either Adhesive Crystallography Seal (Thermo Scientific) or HDClear tape (Duck brand). This gave a total plate thickness of ∼400 µm including the removable UV-film layer. In the second method, a 100 µm thick double-sided adhesive spacer in a 96-well format designed for LCP specific crystallization was attached to polymer film (ZeonorFilm ZF14-013 and ZF14-023; Zeon Europe GmbH, Düsseldorf, Germany) cut by hand to the appropriate size. Liquid was then dispensed, after which the plates were sealed with a top layer of polymer film to complete the sandwich (total thickness 126 µm). The third method used a custom spacer formed from 10 µm thick double-sided tape (Duplocoll 97001; Lohman Tapes, Milton Keynes, England). Apertures forming the crystallization wells were cut in the tape by hand using a hole punch. The tape was then attached to one layer of polymer film to form a platform for liquid deposition in the same manner as the second method. Again the sandwich was completed with a top layer of polymer film attached and sealed by hand (total thickness 36 µm). From here on, this plate type will be referred to as ‘in-house’. Crystallization solutions were dispensed by one of three methods: robotically using either an Echo liquid handler (Labcyte, Sunnyvale, California, USA) or a Mosquito Crystal liquid handler (TTP Labtech, Cambridge, England) or, in the case of the in-house design, by hand using a 10 µl syringe dispenser. Tetragonal lysozyme crystals typically formed within minutes but continued to grow for up to 4 days at 20°C and belonged to space group *P*4_3_2_1_2 (see §[Sec sec3.5]3.5 for further details).

**Figure 3:**
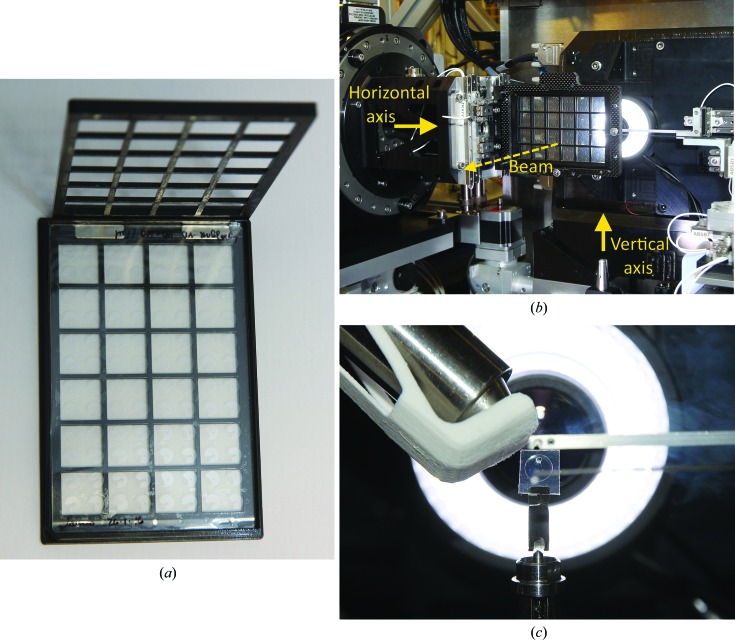
(*a*) A photograph showing a 96-well (8 × 12) TPFS plate placed in an in-house-designed three-dimensional printed adaptor conforming to the SBS format of microtitre plates. The adaptor is in two pieces held together by magnets. (*b*) The plate holder, ready for data collection, mounted on the *in situ* horizontal goniometer of beamline I24 at Diamond Light Source. The vertical goniometer is visible at the bottom of the picture (upward arrow) having been retracted from the sample position. (*c*) A photograph of an isolated well held in a prototype tweezer pin and mounted on the vertical goniometer of beamline I24 at 294 K with the cryoshutter closed; the view is along the beam axis away from the detector.

The TPFS plates were prepared for data collection by mounting the entire 96-well film in a three-dimensional printed holder produced in-house (Fig. 3 ▸ *a*) and using a gonio­meter specific to crystallization plates (Axford *et al.*, 2012 ▸). Alternatively, individual wells were cut out of a whole film using a scalpel and mounted on a SPINE cap using a prototype ‘tweezer’ pin produced by Molecular Dimensions, Newmarket, England (Supplementary Fig. S1 and Fig. 3 ▸ *c*). The tweezer pin is compatible with standard sample vials. Test data were collected on beamline I24 at Diamond Light Source. The beamline has two goniometers: one capable of mounting plates conforming to the SBS format for data collection at room temperature (Fig. 3 ▸ *b*) and the other for the mounting of more conventional loops and from which data could be collected at both room and cryogenic temperatures with the aid of a cryostream (Oxford Cryosystems, Long Hanborough, England) with a cryoshutter on a motorized translation stage (Fig. 3 ▸ *c*). Opening of the cryoshutter allowed rapid cooling in the 100 K gas flow. The diameter of the cryostream flow was ∼7 mm, which was large enough to accommodate the 5 mm diameter of the aperture forming a well of the TPFS. The angle of the cryostream relative to the rotation axis was ∼135° in the vertical plane and ∼5° in the horizontal plane (Fig. 3 ▸ *c*). The unattenuated incident X-ray flux was approximately 1.2 × 10^12^ photons s^−1^ in a beam focus at the sample of 10 × 10 µm and the detector used was a Pilatus3 6M (Dectris, Baden-Dättwil, Switzerland). For the two example data sets described in §[Sec sec3.5]3.5, 900 frames of 0.1° oscillation were collected at 100 frames per second with the beam attenuated to either 20 or 40% of the full flux. Diffraction data were indexed, integrated and scaled automatically with the Diamond MX pipeline software *xia*2 (Winter & McAuley, 2011 ▸) and the resulting processing statistics were assessed.

## 3. Results and discussion   

### 3.1. Crystal growth in TPFS plates   

Lysozyme crystals grew successfully in all three forms of the sandwiches described in §[Sec sec2]2 *via* both the vapour-diffusion and batch methods and examples are presented in Fig. 2 ▸. Vapour-diffusion conditions typically produced smaller numbers of larger crystals.

### 3.2. Comparison of the background scatter from different crystallization platforms   

The background scatter of the TPFS plates was assessed against some current commercial plate types marketed for *in situ* data collection. The Greiner CrystalQuickX plate type represents a ‘traditional’ construction consisting of an injection-moulded tray of cyclic olefin copolymer (COC) with a minimum material thickness below the well of 250 µm and a layer of HDClear sealing tape above it, which is ∼70 µm biaxially orientated polypropylene (BOPP). The MiTeGen *In Situ*-1 plate has an injection-moulded frame but the base of the well is formed by a 100 µm COC film, a thickness that is not easily achievable *via* injection moulding. HDClear sealing tape was again used as a sealing cover. The viewdrop III-formed TPFS plates consist of a 188 µm film but with a removable layer designed to enhance UV-imaging compatibility and an adhesive layer of 150 µm in thickness. HDClear tape was used as the sealing layer for this test. All of the thicknesses reported above are as quoted by the manufacturers.

**Figure 4:**
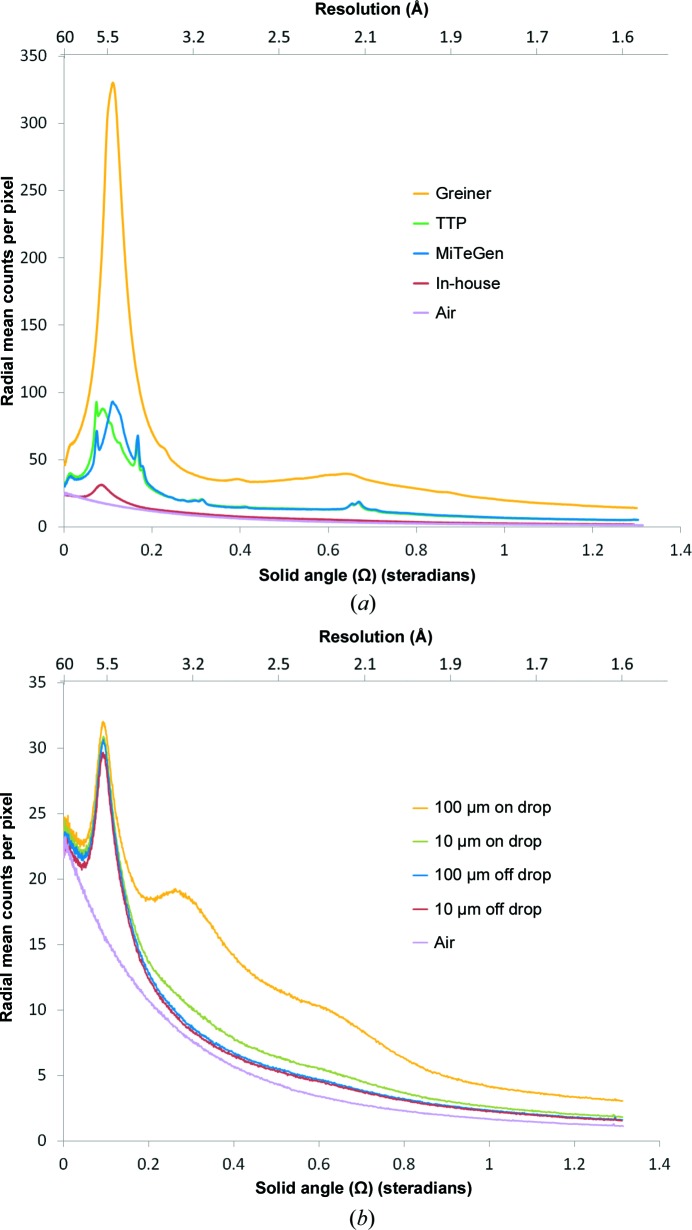
(*a*) A comparison of the background scatter of different plate types. The data are counts per pixel averaged over a constant-resolution ring against solid angle and are normalized to transmittance for each type and result from an exposure of ∼1.2 × 10^11^ photons at 12 800 eV in a beam size of approximately 10 × 10 µm. Plate types shown are Greiner CrystalQuickX, MiTeGen *In Situ*-1, a TPFS formed from a TTPLabtech viewdrop III cover (UV layer removed) (these three plate types are each sealed with HDClear tape) and finally an in-house-constructed TPFS formed from two layers of 13 µm ZeonorFilm with a 10 µm thick adhesive spacer. (*b*) Background scatter comparison using the same methodology as in (*a*) of the 13 µm ZeonorFilm TPFS formed with either a 100 µm spacer or a 10 µm spacer and contrasting measurements taken on and off a water drop. Air-scatter data for comparison give an approximation to ideal loop mounting (less any sample contribution).

The pattern and intensity of the scattering from a material recorded on a detector is a function of the composition of the material and also the path length of the X-rays through the material. Amorphous materials produce a diffuse scattering pattern; more ordered materials can produce sharper rings or spots, which could potentially conceal even strong sample diffraction spots. The scattering characteristics of the different plate types are shown in Fig. 4 ▸(*a*). The in-house TPFS demonstrated by far the lowest overall scattering, with a contribution at many angles (resolutions) approaching that of the bare air path. This was expected since the 26 µm overall path length of the support material is considerably less than the other plate types. The scattering patterns of the MiTeGen *In Situ*-1 and the viewdrop III, both with the same cover tape, were nearly identical, confirming that they were composed of the same material and suggesting that they were of the same thickness.

### 3.3. The background contribution from the drop volume   

Fig. 4 ▸(*b*) shows not only how the film material and its thickness influence the level of background scatter, but also how the thickness of the spacer can affect the amount of scatter since it determines the amount of material within the sandwich. A thinner sandwich was seen to have a reduced background contribution from the drop since the scattering path through the plate is reduced as the liquid volume is spread over a wider area of the well. On-beamline images of larger crystals (Figs. 2 ▸ *b* and 2 ▸ *d*) show how they grew to fill the void between the two layers of film, excluding the solvent volume completely from the beam path; this reduced the background scatter from the solvent to an absolute minimum.

### 3.4. Mounting of TFPS plates for data collection   

The three-dimensional printed holder enabled the thin-film sandwiches to be handled like a conventional SBS-format design for imaging and *in situ* data collection. The *in situ* goniometer allowed an accessible angular range of ∼45° and software-controlled automated motion between wells to facilitate throughput of data collection. The plate holder supported the film when inverted such that no flexing of the film was observed during translations and rotations. When wells were isolated from a larger film, some movement of the contents of the drop was difficult to avoid during the cutting procedure; however, it did not appear to affect the sample integrity. The tweezer pin was found to provide a rigid clamp along the bottom edge of the well which precluded any movement of the isolated section of film.

### 3.5. Data collection from lysozyme crystals   

Fig. 2 ▸(*c*) shows on-beamline views of lysozyme crystals in the in-house TPFS (10 µm thick spacer) held by a tweezer pin on the vertical goniometer. The accessible angular range was explored successfully up to 90° in total (±45°) with data firstly collected from a single crystal over this range at 294 K (Fig. 2 ▸ *b*). The cryostream was then inserted and the cryo­shutter was opened to flash-cool the entire well. Some movement of the sample was then observed as the film cooled, but it stabilized within seconds. The sample was then relocated on the beam axis, offset from the room-temperature data-collection location by approximately double the beam radius to bring in fresh sample, and a second data set was collected. Double the X-ray dose was used for the 100 K data collection since it was anticipated that the higher resolution reflections could be recorded and that radiation damage would not be limiting. Representative statistics for data collection from the same (single) crystal first at 294 K and then cooled to 100 K are shown in Table 1 ▸. In this case the crystal dimensions were approximately 100 × 50 × 10 µm. Whilst in the open cryogenic gas flow, no observable sample motion was induced by the gas flow. At the extremities of the angular range (±45° relative to the gas flow) some slow drifting of the sample was seen. This was attributed to thermally induced contractions in the outer edges of the film as exposure to the gas flow changed slightly, but this motion was not sufficient to move the sample out of the beam. However, since a standard alignment procedure of centring the sample at 0 and 90° could not be performed, there is the potential for some misset to the rotation axis which would bring in fresh sample during the rotation. The processing statistics presented in Table 1 ▸ indicate that the data obtained were of high quality, extending to high resolution and certainly not worse than one would expect from a comparable sample fished and cooled in a conventional manner. The data quality from the plate goniometer matched that of the 294 K pin data, albeit with a lower angular range, limiting completeness. The cryocooling process for the in-house TPFS is illustrated in Supplementary Video S1 captured during data collection. Although transient ice formation was seen within the drop in the annealing step as the well warms, the cooling step is seen to be very rapid and is only indicated by a slight jump in sample position, with no ice formation. As might be expected, sample-cooling effectiveness was found to decline with increasing drop volume (spacer thickness) and increasing film thickness. Crystals in deeper 100 µm spaced sandwiches of 13 µm film, such as those in Fig. 2 ▸(*d*), distanced from the main volume of a drop, cooled effectively without ice formation. Crystals within a large mass of mother liquor were more susceptible to ice formation. On the other hand, ice formation in the viewdrop III-based sandwich could not be completely avoided irrespective of liquid volume, with this attributed to the >100 µm thickness of polymer across which heat must be conducted.

The observation that crystal growth is contained by the volume of the sandwich clearly implies that preferred orientations are induced based on which facet of a growing crystal first aligns with the surface of a film. The fact that 90° of useful data can be collected from samples within an isolated well mitigates this issue to an extent.

At room temperature, microcrystals contained within the TPFS that were not yet attached to either film could be seen to move slowly in convection currents within the sandwich, presumably induced by very slight temperature gradients. However, data could still be collected in a serial manner in the form of stills *via* a raster scan (Fig. 2 ▸ *c*). Once flash-cooled to 100 K any motion ceased and single crystals could be addressed for rotation data collection. Near the edges of a drop and where the drop edge had receded, microcrystals could be seen to be stationary, attached to one of the film layers.

### 3.6. Drop evaporation from thin-film sandwiches   

One issue in moving to thinner polymer films is that the water-permeability of the crystallization platform increases. This results in accelerated drying of the drop relative to more conventional platforms such as a thick injection-moulded tray with thick (>75 µm) sealing tape. The type of polymer used has an effect, with COP/COC and fluorinated ethylene propylene (FEP; including Teflon) being less water-permeable than polyethylene terephthalate (PET; including Mylar): 0.12, 0.17 and 0.5 g mm m^−1^ d^−1^ atm^−1^, respectively, at 25°C (based on material datasheet information). The drop-drying phenomenon can be arrested by creating an external sandwich of glass (Huang *et al.*, 2015 ▸) or plastic which can be sealed using vacuum grease or paraffin film. When exposed to a dry atmosphere of 12% relative humidity, a 150 nl drop of pure water in the thinnest 13 µm ZeonorFilm sandwich was seen to evaporate at a near-constant rate of ∼7 nl h^−1^ monitored over an 18 h period, a worst-case scenario representing only the period on the beamline for data collection. When a second seal was applied, drop drying was greatly arrested, with no obvious change observed by eye to drops over a three-week storage period in an incubator at 18°C. There were also no significant changes to the drop conditions observable during the course of a data-collection session, and the option to flash-cool an individual well provides another method to arrest evaporation. A gradual but controlled evaporation may lead to extended crystal growth and the removal of mother liquor for enhanced *in situ* cryocooling, as clearly illustrated in Fig. 2 ▸(*d*).

## 4. Conclusion   

Sandwiches of thin polymer film can provide a versatile and effective platform for protein crystallization experiments. We have shown how this platform can be applied to soluble protein and in so doing expand its application from the lipidic cubic phase crystallizations of its original conception. Robotic liquid-handling apparatus are perfectly able to dispense to this form of crystallization platform. Alternatively, it is straightforward to dispense by hand to home-made TPFS constructs utilizing low-cost consumables. An additional benefit over more conventional format microtitre plates is that the TPFSs occupy a much smaller volume for storage and initial tests suggest that they are less susceptible to drop movement *via* shaking, which would be advantageous for sample shipping. Early findings indicate that when held in the printed holder the plates are compatible with a Rock Imager 1000 (Formulatrix, Bedford, Massachusetts, USA) for storage and automated imaging. In such an arrangement barcodes could be attached to the plate holder or even printed in a two-dimensional form onto the film if developed commercially.

By moving to thinner films and narrower sandwiches, unwanted X-ray scattering contributions from the plate material and mother liquor are reduced. The method thus becomes optimized for microcrystal investigations on samples that might otherwise be hard to visualize or handle. Such samples, which are relatively common in the early stages of crystallization trials, could easily be lost if conventional fishing were attempted and are likely to prove to be too weakly diffracting to be effectively analysed *in situ* in more traditional crystallization platforms. The ability to collect useful structural information from early hits in crystallization trials can mitigate the need for further rounds of crystallization optimization (Axford *et al.*, 2012 ▸). The demonstration of data collection from the same sample at both room and cryogenic temperatures could prove useful for temperature-dependent structural studies, particularly given the absence of manual handling. The effectiveness of cryocooling in crystallization solutions with different salt or additive solutions to that used here is the subject of ongoing investigation.

Accelerated drop drying is a factor that should be considered when adopting a TPFS for crystallization experiments, and we are undertaking further investigation of the process. In order to control the effects of accelerated drop evaporation, methods such as an additional external glass, plastic or film seal or a humidity-controlled environment can be applied, and we are exploring the performance of different options for long-term storage. Nevertheless, it might be expected that experimenters find that they have to slightly adapt crystallization conditions that have worked successfully in a different crystallization platform. There is the potential, however, to use gradual and controlled evaporation through a thin polymer as another route to crystallization and also to facilitate the removal of mother liquor from a crystal to allow efficient *in situ* flash-cooling. There remains the scope to move to even thinner layers to completely optimize background scatter, but with thinner material typically comes more difficult handling. Alternative materials such as graphene and silicon nitride may be considered but, for the time being, appear to be expensive and complex to manufacture in comparison to the simple methods described here.

## Supplementary Material

Supplementary Figure S1 and description of Supplementary Video.. DOI: 10.1107/S2053230X16004386/nj5248sup1.pdf

Supplementary Video S1.. DOI: 10.1107/S2053230X16004386/nj5248sup2.mp4

## Figures and Tables

**Table 1:** Diffraction data-processing statistics reported by *xia*2 and recorded from a single lysozyme crystal at both 294 and 100 K Values in parentheses are for the highest resolution shell.

	294 K	100 K
Space group	*P*4_3_2_1_2	*P*4_3_2_1_2
Unit-cell parameters (Å, °)	*a* = *b* = 78.56, *c* = 38.51, α = β = γ = 90	*a* = *b* = 77.55, *c* = 38.10, α = β = γ = 90
Resolution (Å)	39.28–1.45 (1.49–1.45)	38.10–1.15 (1.18–1.15)
*R*_p.i.m._	0.038 (0.66)	0.033 (0.025)
*R*_meas_	0.100 (1.369)	0.081 (0.700)
〈*I*/σ(*I*)〉	9.0 (1.2)	10.2 (1.1)
Mosaicity† (°)	0.039	0.477
Completeness (%)	96.5 (95.1)	85.9 (39.5‡)
Multiplicity	4.9 (3.6)	3.3 (3.1)
CC_1/2_	0.997 (0.545)	0.995 (0.543)

† As reported by *XDS*.

‡ The lower completeness for the 100 K data set is owing to the highest resolution reflections only being recorded in the corners of the detector.

## References

[bb1] Aller, P. *et al.* (2015). *Structural Proteomics: High-Throughput Methods*, edited by R. J. Owens, pp. 233–253. New York: Springer.

[bb2] Axford, D., Foadi, J., Hu, N.-J., Choudhury, H. G., Iwata, S., Beis, K., Evans, G. & Alguel, Y. (2015). *Acta Cryst.* D**71**, 1228–1237. 10.1107/S139900471500423X PMC4461203 26057664

[bb3] Axford, D. *et al.* (2012). *Acta Cryst.* D**68**, 592–600. 10.1107/S0907444912006749 PMC4791750 22525757

[bb4] Bingel-Erlenmeyer, R., Olieric, V., Grimshaw, J. P. A., Gabadinho, J., Wang, X., Ebner, S. G., Isenegger, A., Schneider, R., Schneider, J., Glettig, W., Pradervand, C., Panepucci, E. H., Tomizaki, T., Wang, M. & Schulze-Briese, C. (2011). *Cryst. Growth Des.* **11**, 916–923.

[bb5] Caffrey, M. (2015). *Acta Cryst.* F**71**, 3–18. 10.1107/S2053230X14026843 PMC4304740 25615961

[bb6] Cipriani, F., Röwer, M., Landret, C., Zander, U., Felisaz, F. & Márquez, J. A. (2012). *Acta Cryst.* D**68**, 1393–1399. 10.1107/S0907444912031459 22993093

[bb8] Heidari Khajepour, M. Y., Vernede, X., Cobessi, D., Lebrette, H., Rogues, P., Terrien, M., Berzin, C. & Ferrer, J.-L. (2013). *Acta Cryst.* D**69**, 381–387. 10.1107/S0907444912048019 23519413

[bb9] Huang, C.-Y., Olieric, V., Ma, P., Panepucci, E., Diederichs, K., Wang, M. & Caffrey, M. (2015). *Acta Cryst.* D**71**, 1238–1256. 10.1107/S1399004715005210 PMC4461204 26057665

[bb17] Huang, C.-Y., Olieric, V., Ma, P., Howe, N., Vogeley, L., Liu, X., Warshamanage, R., Weinert, T., Panepucci, E., Kobilka, B., Diederichs, K., Wang, M. & Caffrey, M. (2016). *Acta Cryst.* D**72**, 93–112. 10.1107/S2059798315021683 PMC4756617 26894538

[bb10] Kisselman, G., Qiu, W., Romanov, V., Thompson, C. M., Lam, R., Battaile, K. P., Pai, E. F. & Chirgadze, N. Y. (2011). *Acta Cryst.* D**67**, 533–539. 10.1107/S0907444911011589 PMC3107051 21636893

[bb11] Maire, A. le, Gelin, M., Pochet, S., Hoh, F., Pirocchi, M., Guichou, J.-F., Ferrer, J.-L. & Labesse, G. (2011). *Acta Cryst.* D**67**, 747–755. 10.1107/S0907444911023249 21904027

[bb12] Murray, T. D., Lyubimov, A. Y., Ogata, C. M., Vo, H., Uervirojnangkoorn, M., Brunger, A. T. & Berger, J. M. (2015). *Acta Cryst.* D**71**, 1987–1997. 10.1107/S1399004715015011 PMC4601365 26457423

[bb13] Roedig, P., Vartiainen, I., Duman, R., Panneerselvam, S., Stübe, N., Lorbeer, O., Warmer, M., Sutton, G., Stuart, D. I., Weckert, E., David, C., Wagner, A. & Meents, A. (2015). *Sci. Rep.* **5**, 10451. 10.1038/srep10451 PMC4448500 26022615

[bb14] Wagner, A., Duman, R., Stevens, B. & Ward, A. (2013). *Acta Cryst.* D**69**, 1297–1302. 10.1107/S090744491300958X PMC3689533 23793156

[bb15] Wang, X. *et al.* (2012). *Nature Struct. Mol. Biol.* **19**, 424–429. 10.1038/nsmb.2255 PMC3378640 22388738

[bb16] Winter, G. & McAuley, K. E. (2011). *Methods*, **55**, 81–93. 10.1016/j.ymeth.2011.06.010 21763424

