# Supplementary material for: Applications of thin-film sandwich crystallization platforms
Source: Acta Crystallogr F Struct Biol Commun. 2016 Mar 24;72(Pt 4):313–9. doi: 10.1107/S2053230X16004386 (PMC4822989; doi:10.1107/S2053230X16004386)
Supplement: Supplementary file 1 [file f-72-00313-sup1.pdf]

STRUCTURAL BIOLOGY COMMUNICATIONS

Volume 72 (2016)

Supporting information for article:

Applications of thin-film sandwich crystallization platforms

Danny Axford, Pierre Aller, Juan Sanchez-Weatherby and James Sandy

The video shows a live demonstration of *in situ* cryo-cooling and warming to room temperature as viewed through the on-axis viewing system presented in the GDA user interface software on the beamline. In this case the sample is a TPFS well formed from two layers of 13 µm polymer and 10 µm thick spacer. The video starts with the well at 100 K having been manually mounted into the open flow of the cryostream (see **Error! Reference source not found.** (c)). At t = 3s the cryo shutter is closed (the mouse cursor depresses the button in the software window) and the well warms to room temperature, transient ice formation can be seen in the drop volume on the left of the view. Gentle movement of the contents of the drop confirm that it is again liquid. Then at t = 23s the cryo-shutter is opened and the well rapidly cooled. Contents of the drop can be seen to undergo a near instantaneous 'jump' as the surrounding liquid becomes solid and there is no indication of any ice formation (confirmed by X ray measurements). This cycle could be repeated consistently many times.

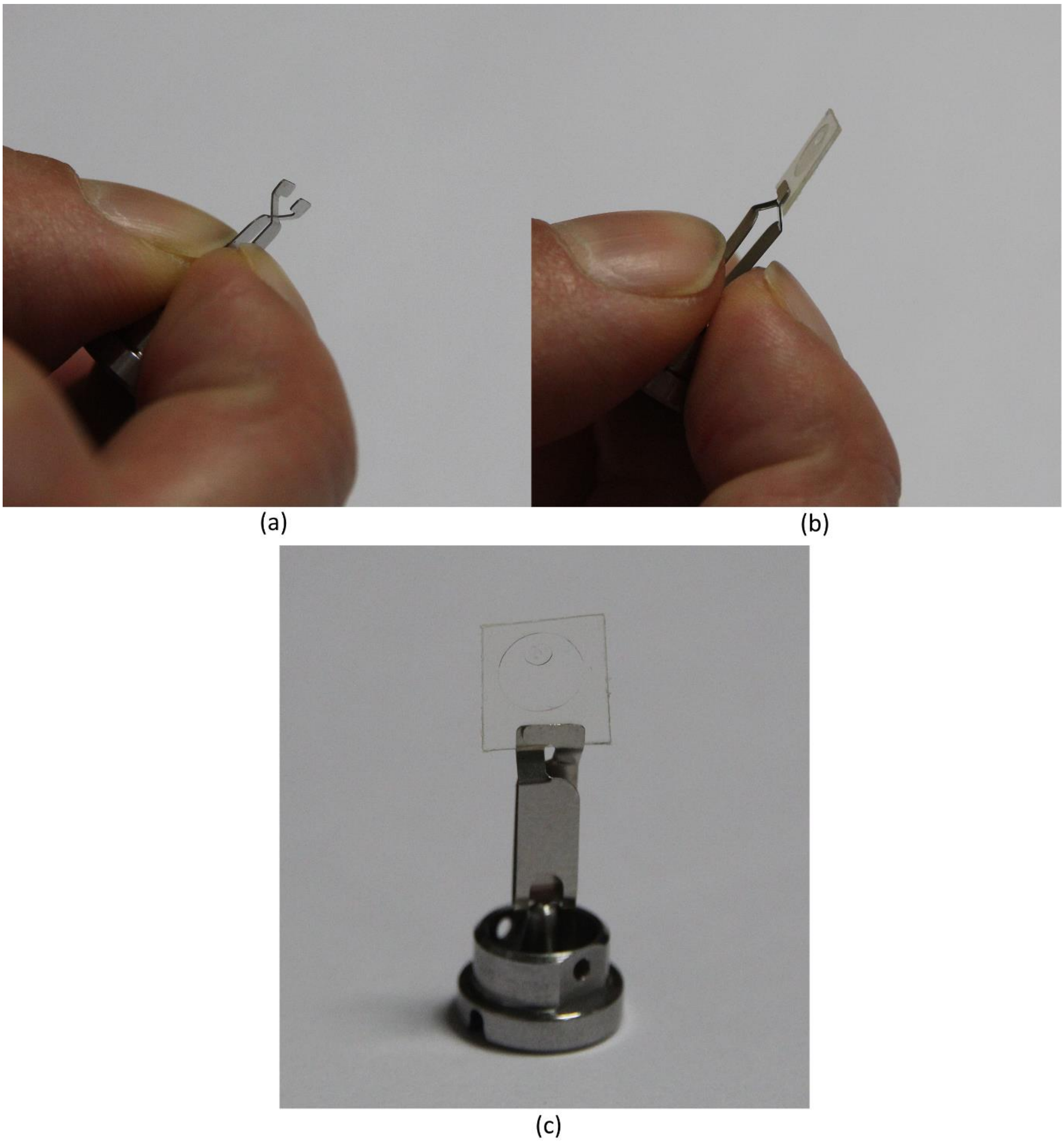

Figure S1 Close up images showing (a) opening of tweezer pin (Diffrax™ sample holder spring clip), (b) closed around an isolated well cut by scalpel from a 96 well film. (c) Isolated well with drop ready for mounting on a goniometer.
